# Foam Sclerotherapy for Conjunctival Inclusion Cyst Post Evisceration

**DOI:** 10.7759/cureus.41570

**Published:** 2023-07-08

**Authors:** Rwituja Thomas, Ashok Grover

**Affiliations:** 1 Oculoplastic Surgery, Vision Eye Centre, New Delhi, IND; 2 Ophthalmology, Sir Ganga Ram Hospital, New Delhi, IND

**Keywords:** orbital implant, evisceration, sodium tetradecyl sulfate, orbital cyst, foam sclerotherapy

## Abstract

Orbital conjunctival epithelial cysts have traditionally been excised, with the risk of leaving behind remnants that may result in recurrences. We present an 18-year-old male who complained of a poorly retained prosthesis three years after a primary evisceration and polymethylmethacrylate (PMMA) ball implant. We performed cyst aspiration and injection foam sclerotherapy for the cyst, which resolved completely in six weeks, allowing the prosthesis to be retained comfortably. Aspiration and injection of sclerosing agents may result in the collapse of the cyst along with fibrosis of their walls with obliteration of the lumen, resulting in complete resolution.

## Introduction

Conjunctival epithelial inclusion cysts of the orbit following evisceration are uncommon; however, there have been several modalities of treatment used to manage them. In an anophthalmic socket, these cysts can result in an ill-fitting ocular prosthesis due to the rise in surface area or migration of the orbital implant. The patient might suffer from the inability to retain the prosthesis, and this is usually when the patient presents, resulting in poor cosmesis [1]. These cysts can be dealt with using several methods, such as complete excision and marsupialization, sometimes aided by the use of fibrin glue injection. They can be injected with sclerosing agents such as sodium tetradecyl sulfate (STS), absolute alcohol, trichloroacetic acid, and bleomycin [2]. As it may be difficult to entirely excise these cysts without leaving any residual remnants that can cause recurrence, it would be wise to master the injection of sclerosing agents to achieve a good result. Here, we report an orbital inclusion cyst in an anophthalmic socket following evisceration with primary polymethylmethacrylate (PMMA) ball implantation treated by foam sclerotherapy. An extensive literature review reveals that foam sclerotherapy has never been reported in an orbital conjunctival cyst in an eviscerated socket.

## Case presentation

**Figure 1:**
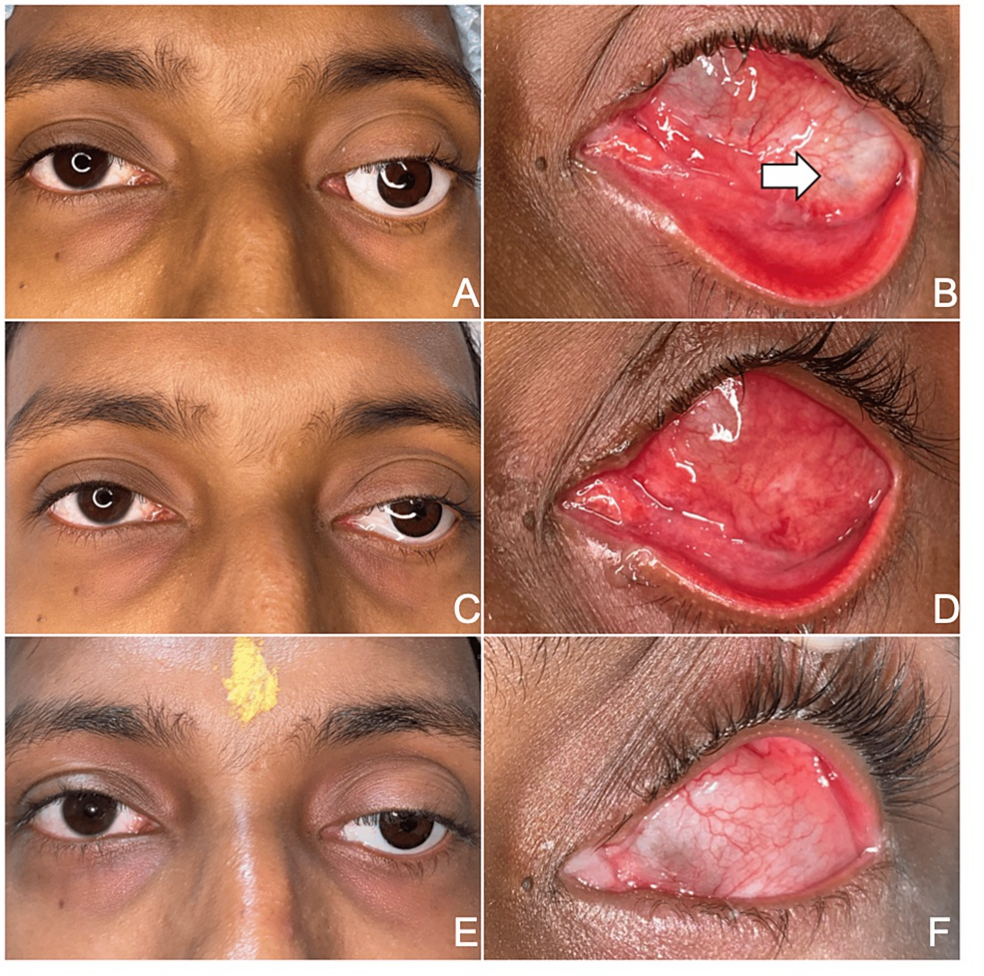
Sequential improvement post injection with and without prosthesis A: External photograph of the patient showing an ill-fitting prosthesis. B: White arrow showing the position of the conjunctival cyst. C, D: One-week external photograph showing partial resolution with the improved fitting of the prosthesis. E, F: Six-week external photograph showing complete resolution with the good fitting of the prosthesis

An 18-year-old Indian male presented to us with complaints of displacement of his custom ocular prosthesis (Figure 1a) and occasionally the inability to retain the prosthesis within the socket. He had undergone an evisceration along with primary placement of a PMMA implant of the left eye four years prior due to an anterior staphyloma. He described a gradual displacement of his prosthesis over the past nine months. On examination, it was found that he had a conjunctival cyst within the orbit (Figure 1b), which had resulted in the orbital implant being pushed superotemporally, thus explaining the resultant prosthesis displacement. One week post the injection, there was a reduction in the proptosis caused by the cyst, and the patient was able to retain the prosthesis (Figure 1c, 1d). Six weeks after the procedure, the cyst had resolved completely, and the prosthesis was well placed (Figure 1e, 1f).

**Figure 2:**
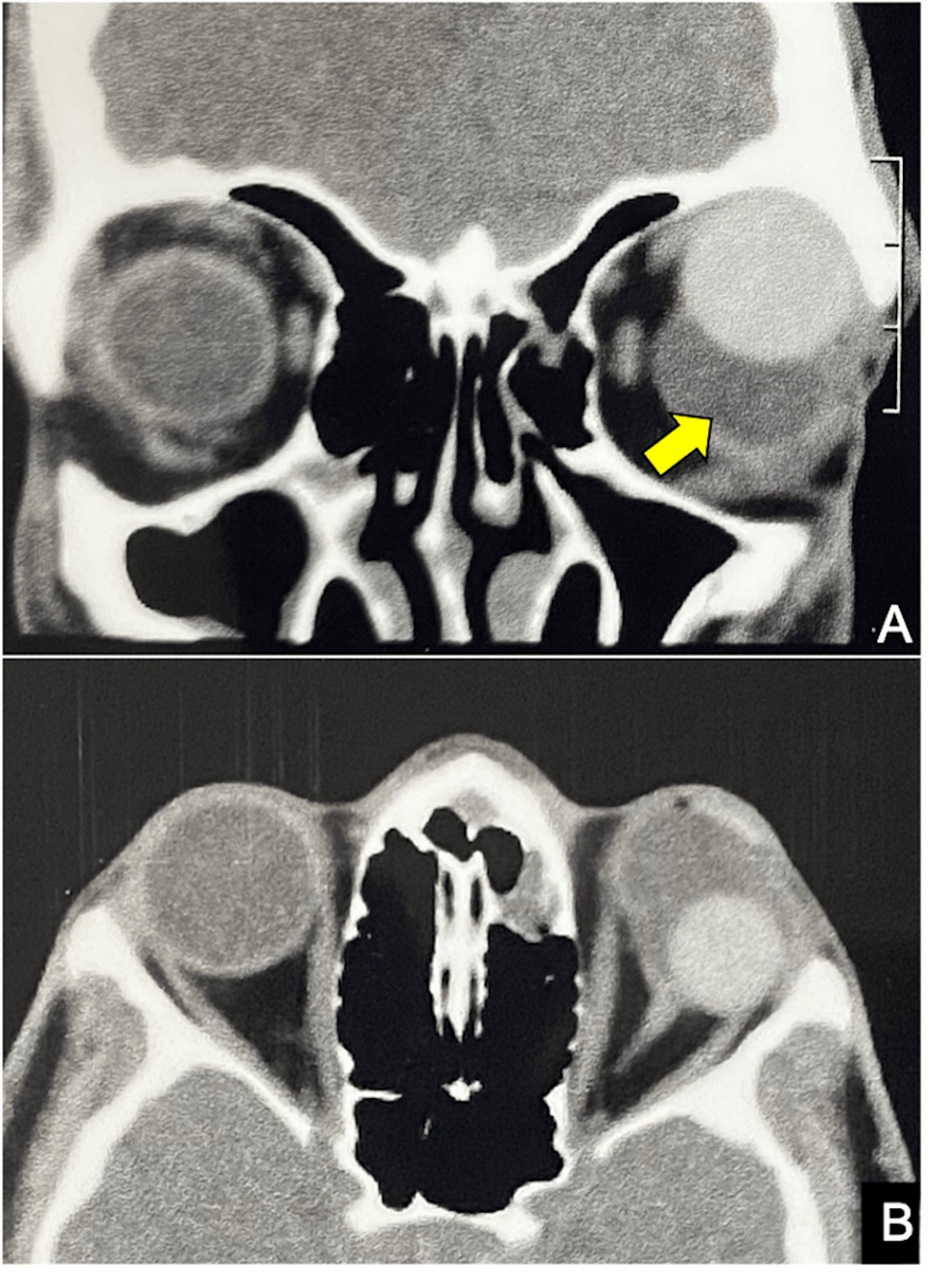
Plain CT scan orbits CT scan image with coronal and sagittal cuts demonstrating the location of the cyst (yellow arrow)

CT of the orbit revealed an orbital fluid-filled cyst that had shifted the PMMA implant upward (Figure 2a, 2b) and consequently increased the surface area of the socket too.

**Figure 3:**
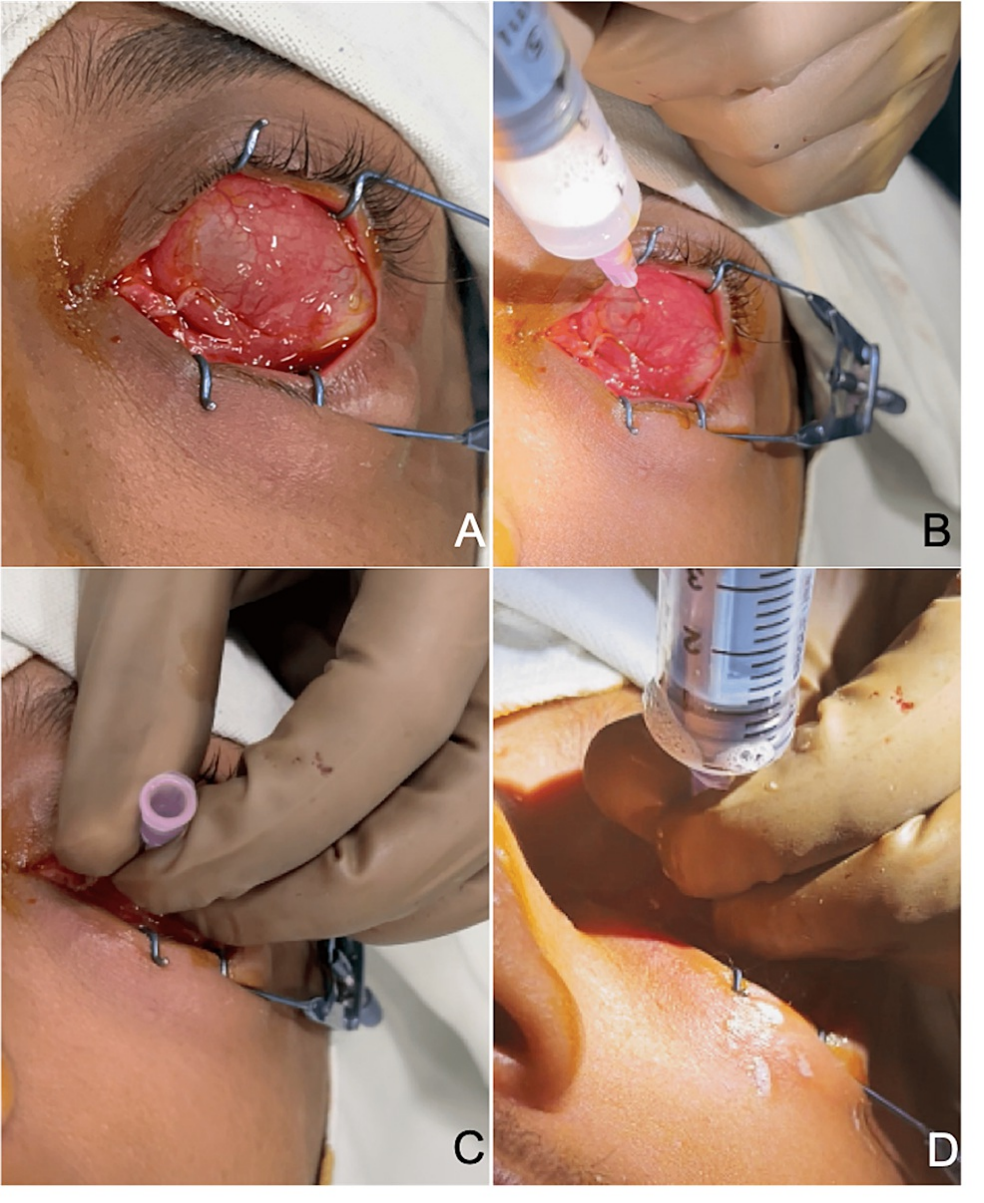
Intraoperative photographs A: Full area of cyst exposed with a speculum. B: Cyst aspirated with a 23 gauge needle and syringe. C: Syringe with aspirate removed. D: Injection of foamed STS

We then proceeded to perform injection sclerotherapy for the patient. In the supine position, with topical anesthetic instilled, the area was painted and draped, and a speculum was placed on the side of the anophthalmic socket (Figure 3a). With the help of a 23 gauge needle and 10 ml syringe, the needle was inserted into the highest point of the cyst and aspirated (Figure 3b). Once all the fluid was removed, the syringe was replaced (Figure 3c) with a syringe containing foamed STS solution (30mg/ml) and injected into the hollow cyst (Figure 3d).

In the first 48 hours after the procedure, the patient may experience minimal discomfort due to transient inflammation prior to the onset of fibrosis. The patient preferred the option of prosthesis modification for the management of his left upper lid ptosis.

## Discussion

Foam sclerotherapy is an inexpensive and easily performed procedure that has been used for periorbital dermoid cysts [3] and veno-lymphatic malformations of the eyelid and anterior orbit [4] with good results. Conjunctival cysts that occur primarily after evisceration and implant are rare, and they usually occur due to a secondary cause such as implantation of the conjunctiva deeper or in case of complex surgery. Complete excision is complicated by thin walls of the cyst that may rupture easily [2]. Thus, it is important that one is able to perform sclerotherapy which, in most cases, works well in the long term. The advantage of this quick and simple procedure is that it can be repeated if needed in case of recurrence or incomplete resolution. If the cyst recurs multiple times, surgical excision with careful dissection and removal of the capsule may be warranted. STS works predictably due to its surfactant nature by cyst wall fibrosis and collapse of the cyst lumen. Patients can be followed up with repeat CT scans of the orbit as necessary.

## Conclusions

To summarize, this is a rare presentation of a conjunctival epithelial cyst after primary orbital implantation following evisceration. Imaging can help to localize the exact position and depth of the posterior wall of the cyst to plan cyst aspiration. STS is an effective treatment as a foam sclerosant for a minimally invasive solution for the management of these cysts.
